# Efficacy of point-of-care thromboelastography 6s to evaluate platelet function in a patient with pseudothrombocytopenia undergoing cardiopulmonary bypass: a case report

**DOI:** 10.1186/s40981-022-00496-6

**Published:** 2022-01-25

**Authors:** Yoshihiko Chiba, Yuji Otsuka, Alan Kawarai Lefor, Masamitsu Sanui

**Affiliations:** 1 Department of Anesthesiology and Critical Care Medicine, Jichi Medical University Saitama Medical Center, 1-847, Amanuma, Omiya, Saitama-City, Saitama, 330-8503 Japan; 2 Department of Surgery, Jichi Medical University, 1-3311, Yakushiji, Shimotsuke-city, Tochigi, 329-0498 Japan

**Keywords:** Pseudo-thrombocytopenia, Thromboelastography 6s, Transfusion

## Abstract

**Background:**

Pseudothrombocytopenia is a phenomenon caused by in vitro platelet aggregation induced by anticoagulants contained in blood sampling tubes. Thromboelastography (TEG) 6s is a common point-of-care viscoelastic test to assess intraoperative coagulation status, which may reduce the need for blood transfusions. The reliability and usefulness of TEG6s for patients with pseudothrombocytopenia has not been established.

**Case presentation:**

We present a patient with pseudothrombocytopenia, who underwent suprarenal abdominal aortic aneurysm repair under cardiopulmonary bypass. At the beginning of surgery, TEG6s with citrated blood sampling showed that the results were unaffected by the disease. After completion of the aortic repair and the administration of protamine, maximum amplitude of TEG6s and adequate hemostasis were achieved without platelet transfusions, while standard laboratory tests showed thrombocytopenia.

**Conclusions:**

In the present patient who underwent cardiopulmonary bypass, TEG6s may have contributed to a reliable and useful assessment of coagulation and hemostatic status avoiding unnecessary platelet transfusions.

## Background

Pseudothrombocytopenia is characterized by in vitro platelet aggregation resulting in spuriously low platelet counts by automated analyzers [1]. In patients with pseudothrombocytopenia, platelet aggregation develops over time in the presence of anticoagulants such as ethylenediaminetetraacetic acid (EDTA), acid citrate dextrose (ACD), and sodium heparin used for the common laboratory analysis [2]. Therefore, a peripheral blood smear is required to accurately assess the platelet count. During surgery using cardiopulmonary bypass, however, such a time-consuming test is not practical, and the decision for platelet transfusion may depend solely on clinical judgment.

Thromboelastography 6s (TEG6s, Haemonetics, Braintree, MA, USA) system is a point-of-care test, which can evaluate whole blood viscoelastic properties and the hemostatic process at the bedside, facilitating personalized transfusion strategies during cardiovascular surgery [3]. However, TEG6s requires citrated blood sampling, which may develop in vitro platelet agglutination and alter the results. For this reason, the reliability and usefulness of TEG6s for the evaluation of hemostasis in patients with citrate-associated pseudothrombocytopenia is unclear.

We used TEG6s for a patient with citrate-associated pseudothrombocytopenia during abdominal aortic aneurysm repair with cardiopulmonary bypass, based on the hypothesis that if TEG analysis is started immediately after blood sampling, the effect of platelet agglutination may be minimized. Written informed consent was obtained.

## Case presentation

**Fig. 1:**
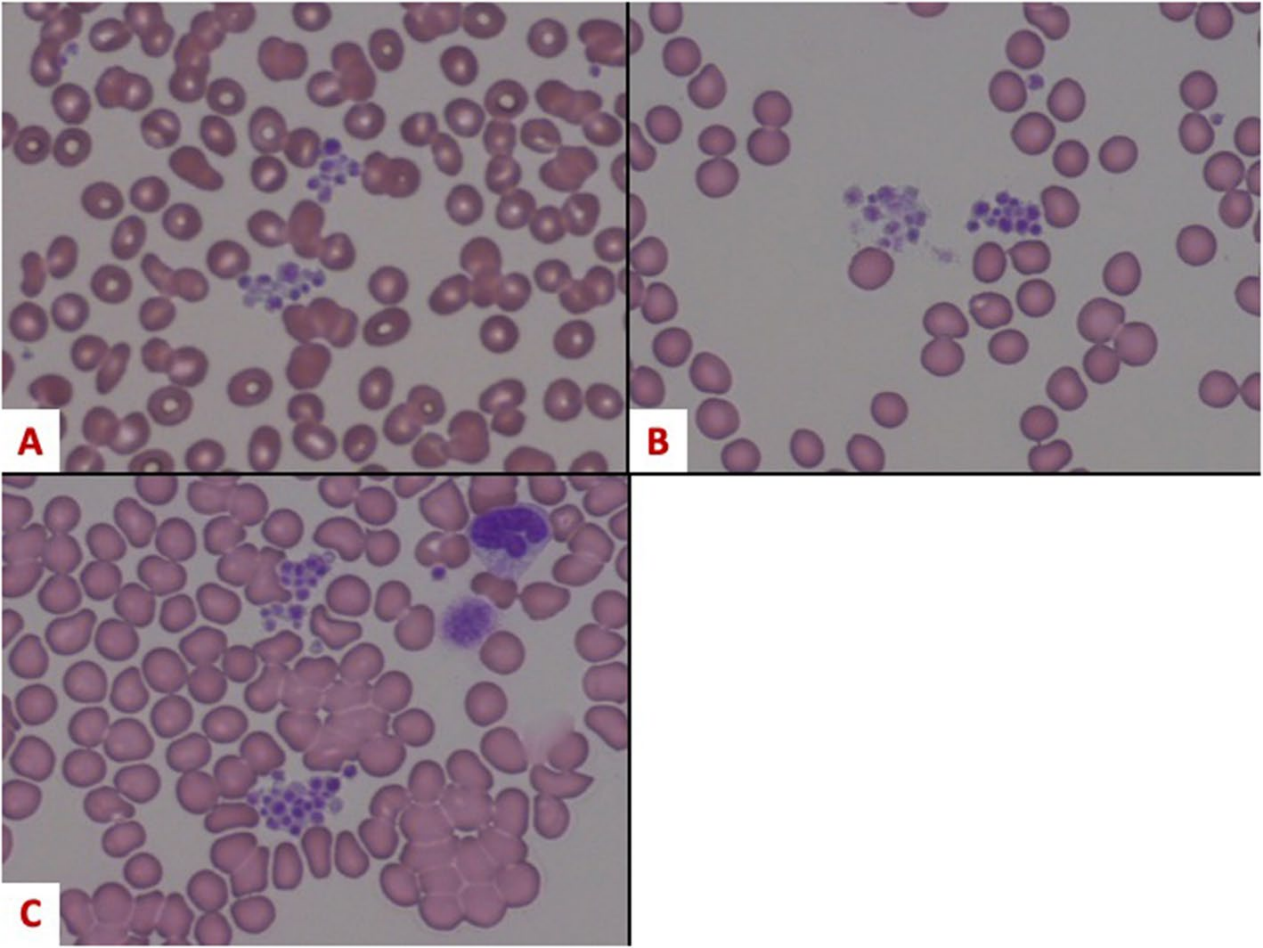
Aggregation of platelets in specimen tubes collected. The preoperative peripheral blood smear showed platelet agglutination in all specimen tubes collected (**A** ethylenediaminetetraacetic acid, **B** acid citrate dextrose, **C** heparin)

An 83-year-old man presented with worsening back pain. Computed tomography scan showed suprarenal, a 58-mm saccular abdominal aortic aneurysm with no evidence of rupture. The patient was scheduled for a semi-urgent surgical repair of the aorta. Initial laboratory tests were normal except for thrombocytopenia (< 4.9 × 10^9^/L, Table 1) determined by a routine automated analyzer. He had no history of bleeding tendencies and was not taking medications associated with thrombocytopenia. No petechial or mucosal bleeding was noted. Bleeding time, International Normalized Ratio of prothrombin time, and activated partial thromboplastin time were all normal. Thrombocytopenia was found not only in the specimen in the EDTA containing tube, but also in tubes containing ACD and sodium heparin. The peripheral blood smear showed platelet agglutination in all specimen tubes collected (Fig. 1). The platelet count increased (11.7 × 10^9^/L, Table 1) with rapid blood test sampling. Antibody tests showed no specific results, and further investigation revealed high immunoglobulin M levels. Bone marrow aspiration was deferred in consideration of the invasiveness of the procedure for a patient with an aortic aneurysm. Based on the results of the tests, clinical evaluation and the peripheral blood smear, the diagnosis of idiopathic pseudothrombocytopenia was made.

**Table 1:** Perioperative laboratory tests and TEG6s results at three time points

	Preoperative	During cardiopulmonary bypass	After protamine administration
Platelet count(× 10^9^/L)			
EDTA	4.9	3.5	3.7
ACD	8.8	–	–
Rapid test from blood sampling (EDTA)	11.7	–	––
Rapid test from blood sampling (heparin)	10.4	–	
Fibrinogen (mg/dL)	369	211	226
Hemoglobin (g/dL)	10.9	6.7–8.2	8.4
Activated clotting time	103	427–700	111
Maximum amplitude of rapid TEG (mm, normal range: 52–70)	66.5	-	57.6
Amplitude at 10 min of rapid TEG (mm, normal range: 44–67)	63.1		48.8
Alpha angle of rapid TEG (degrees, normal range: 60–78)	76.2	-	69.7
Maximum amplitude of citrated functional fibrinogen (mm, normal range: 15–32)	27.6	-	20.0

*Abbreviation*s: *TEG* thromboelastography, *EDTA* ethylenediaminetetraacetic acid, *ACD* acid citrate dextrose

At the beginning of the operation, blood was collected in tubes containing ACD and was immediately examined by a bedside TEG6s analyzer. Coagulation form curves and all values displayed in the TEG6s analyzer were normal. We decided to use TEG6s, especially the maximum amplitude of RapidTEG of TEG6s tests, as a reference for hemostatic and coagulation function during surgery. The activated clotting time determined at the same time was normal (103 s, Table 1). To perform supra-superior mesenteric arterial aortic cross clamping, a left lateral thoracotomy was performed. After administration of 14,000 U heparin (300 U/kg) according to the institutional protocol, activated clotting time was prolonged to 516 s. The right femoral artery and vein were then cannulated, and to perfuse the abdominal viscera, cardiopulmonary bypass was used during the aneurysm repair. Activated clotting time was appropriately maintained above 400 s with additional heparin administration as appropriate (Table 1). The surgery was performed under normothermic conditions, and only one pack of red blood cells (280 mL) was transfused upon initiating cardiopulmonary bypass.

After completion of the aortic repair and cessation of bypass, 200 mg of protamine was administered, based on a calculation of the amount of heparin administered. The activated clotting time returned to 111 s (Table 1), and TEG6s was reevaluated. The maximum amplitude of RapidTEG and citrated functional fibrinogen showed that adequate coagulation and hemostatic condition was derived (Table 1). Bleeding was easily controlled without transfusion of platelets or fresh frozen plasma, although standard laboratory tests showed thrombocytopenia (3.7 × 10^9^/L, Table 1). Postoperatively, the patient was discharged from the hospital without complications, while the platelet count remains low by routine testing.

## Discussion

We present a patient with pseudothrombocytopenia undergoing abdominal aortic aneurysm repair under cardiopulmonary bypass, for whom TEG6s was used to assess intraoperative coagulation and hemostatic function. Platelet aggregation was found in preoperative sampling tubes containing ACD, which was also expected to occur in ACD containing tubes to examine TEG6s. However, the intraoperative results of TEG6s were compatible with results in patients who have normal platelet function, and therefore, transfusion of platelet was avoided. TEG6s may have a role for assessing coagulation and hemostatic function during cardiovascular surgery. Successful use of TEG5000 during coronary bypass surgery for a patient with citrate associated pseudothrombocytopenia was reported [4], but non-citrated blood samples were used in this case. To the best of our knowledge, this is the first case report using TEG6s in a patient with pseudothrombocytopenia.

The incidence of pseudothrombocytopenia in adults is 0.9% to 2%. Pseudothrombocytopenia may be related to malignancy, myeloma, autoimmune disease, liver disease, cardiovascular disease, sepsis, and the use of certain medications [5]. Although the mechanism is not fully understood, the presence of anticoagulant-dependent anti-platelet autoantibodies may induce platelet clumping [2], which is frequently observed in EDTA containing blood samples. Other anticoagulants (citrate, heparin, and oxalate) have also been implicated in the disease, while in 52% of the cases, no apparent causes are identified [6]. To determine the actual platelet count, examination of a peripheral blood smear is required. For intraoperative assessment of the platelet count, using non-agglutinating blood collection tubes is recommended [7]. In this patient, platelet aggregation developed in all blood sample tubes for preoperative evaluation, and platelet aggregation had been expected with citrated blood samples for intraoperative TEG6s.

Four different assays can run simultaneously on the TEG6s within 10 min. RapidTEG, one of the four assays, is an accelerated assay with tissue factor and kaolin for activation of both extrinsic and intrinsic coagulation pathways. In these assays, the maximum amplitude is the point at which the clotting intensity is maximized, reflecting eventual maximal platelet-fibrin interaction via the GPIIb/IIIa receptor. The blood is pipetted into TEG6s cartridges, which accept either citrated or heparinized blood samples. In this patient, citrate-related antibodies against platelet membranes might have been present in the blood samples used for TEG6s, and the results of maximum amplitude might have been affected. However, the results were compatible with results in people with normal platelet function, while the platelet count was simultaneously low when measured by a standard laboratory analyzer.

For patients with pseudothrombocytopenia, in vitro platelet agglutination may progress over time [2]. In this patient, the preoperative platelet count measured by the rapid test was higher than by the standard EDTA or ACD tests (Table 1), suggesting time-dependent platelet aggregation. We performed the TEG6s test expeditiously after blood collection, and therefore, the effect of platelet aggregation on the results of TEG6s was minimized.

Among four different assays of the TEG6s, the citrated functional fibrinogen assay, a method to quantify fibrin polymerization, may not be reliable since the effects of anticoagulant-dependent anti-platelet autoantibodies on GIIb/IIIa inhibitor or GPIIb/IIIa receptors are unclear. In this patient, the maximum amplitude of the citrated functional fibrinogen and serum fibrinogen levels after protamine administration showed adequate coagulation status (Table 1). Adequate hemostasis was also obtained at the operating field. These results suggest that significant platelet agglutination was avoided by expeditious TEG analysis or that no significant interaction was present between the autoantibodies and GIIb/IIIa inhibitors/receptors.

By performing TEG6s analysis, surgical repair of an abdominal aortic aneurysm under cardiopulmonary bypass was successfully performed for a patient with pseudothrombocytopenia. During the surgery, coagulation and hemostatic function evaluated by TEG6s was consistent with clinical findings, not with the platelet count by standard laboratory testing. As a point-of-care test, prompt TEG analysis may have avoided significant citrate-associated platelet aggregation. The reliability and usefulness of TEG6s for patients with pseudothrombocytopenia needs further evaluation with a larger sample of patients undergoing surgery with an associated elevated bleeding risk.

## Data Availability

Not applicable.

## Abbreviations

TEG: Thromboelastography
EDTA: Ethylenediaminetetraacetic acid
ACD: Acid citrate dextrose
